# Interpretable machine learning model for early prediction of 28-day mortality in ICU patients with sepsis-induced coagulopathy: development and validation

**DOI:** 10.1186/s40001-023-01593-7

**Published:** 2024-01-03

**Authors:** Shu Zhou, Zongqing Lu, Yu Liu, Minjie Wang, Wuming Zhou, Xuanxuan Cui, Jin Zhang, Wenyan Xiao, Tianfeng Hua, Huaqing Zhu, Min Yang

**Affiliations:** 1 The 2nd Department of Intensive Care Unit, the Second Affiliated Hospital of Anhui Medical University, Hefei, 230601 Anhui People’s Republic of China; 2 The Laboratory of Cardiopulmonary Resuscitation and Critical Care Medicine, the Second Affiliated Hospital of Anhui Medical University, Hefei, 230601 Anhui People’s Republic of China; 3 Emergency Internal Medicine, the Second Affiliated Hospital of Anhui Medical University, Hefei, 230601 Anhui People’s Republic of China; 4 Key Laboratory of Intelligent Computing and Signal Processing, Anhui University, Ministry of Education, Hefei, 230601 Anhui People’s Republic of China; 5 Laboratory of Molecular Biology and Department of Biochemistry, Anhui Medical University, Hefei, 230022 Anhui People’s Republic of China

**Keywords:** Sepsis induced coagulopathy, Gradient boosting decision tree, Machine learning, Shapley additive explanations, Local interpretable model-agnostic explanations

## Abstract

**Objective:**

Sepsis-induced coagulopathy (SIC) is extremely common in individuals with sepsis, significantly associated with poor outcomes. This study attempted to develop an interpretable and generalizable machine learning (ML) model for early predicting the risk of 28-day death in patients with SIC.

**Methods:**

In this retrospective cohort study, we extracted SIC patients from the Medical Information Mart for Intensive Care III (MIMIC-III), MIMIC-IV, and eICU-CRD database according to Toshiaki Iba's scale. And the overlapping in the MIMIC-IV was excluded for this study. Afterward, only the MIMIC-III cohort was randomly divided into the training set, and the internal validation set according to the ratio of 7:3, while the MIMIC-IV and eICU-CRD databases were considered the external validation sets. The predictive factors for 28-day mortality of SIC patients were determined using recursive feature elimination combined with tenfold cross-validation (RFECV). Then, we constructed models using ML algorithms. Multiple metrics were used for evaluation of performance of the models, including the area under the receiver operating characteristic curve (AUROC), area under the precision recall curve (AUPRC), accuracy, sensitivity, specificity, negative predictive value, positive predictive value, recall, and F1 score. Finally, Shapley Additive Explanations (SHAP), Local Interpretable Model-Agnostic Explanations (LIME) were employed to provide a reasonable interpretation for the prediction results.

**Results:**

A total of 3280, 2798, and 1668 SIC patients were screened from MIMIC-III, MIMIC-IV, and eICU-CRD databases, respectively. Seventeen features were selected to construct ML prediction models. XGBoost had the best performance in predicting the 28-day mortality of SIC patients, with AUC of 0.828, 0.913 and 0.923, the AUPRC of 0.807, 0.796 and 0.921, the accuracy of 0.785, 0.885 and 0.891, the F_1_ scores were 0.63, 0.69 and 0.70 in MIMIC-III (internal validation set), MIMIC-IV, and eICU-CRD databases. The importance ranking and SHAP analyses showed that initial SOFA score, red blood cell distribution width (RDW), and age were the top three critical features in the XGBoost model.

**Conclusions:**

We developed an optimal and explainable ML model to predict the risk of 28-day death of SIC patients 28-day death risk. Compared with conventional scoring systems, the XGBoost model performed better. The model established will have the potential to improve the level of clinical practice for SIC patients.

**Graphical Abstract:**

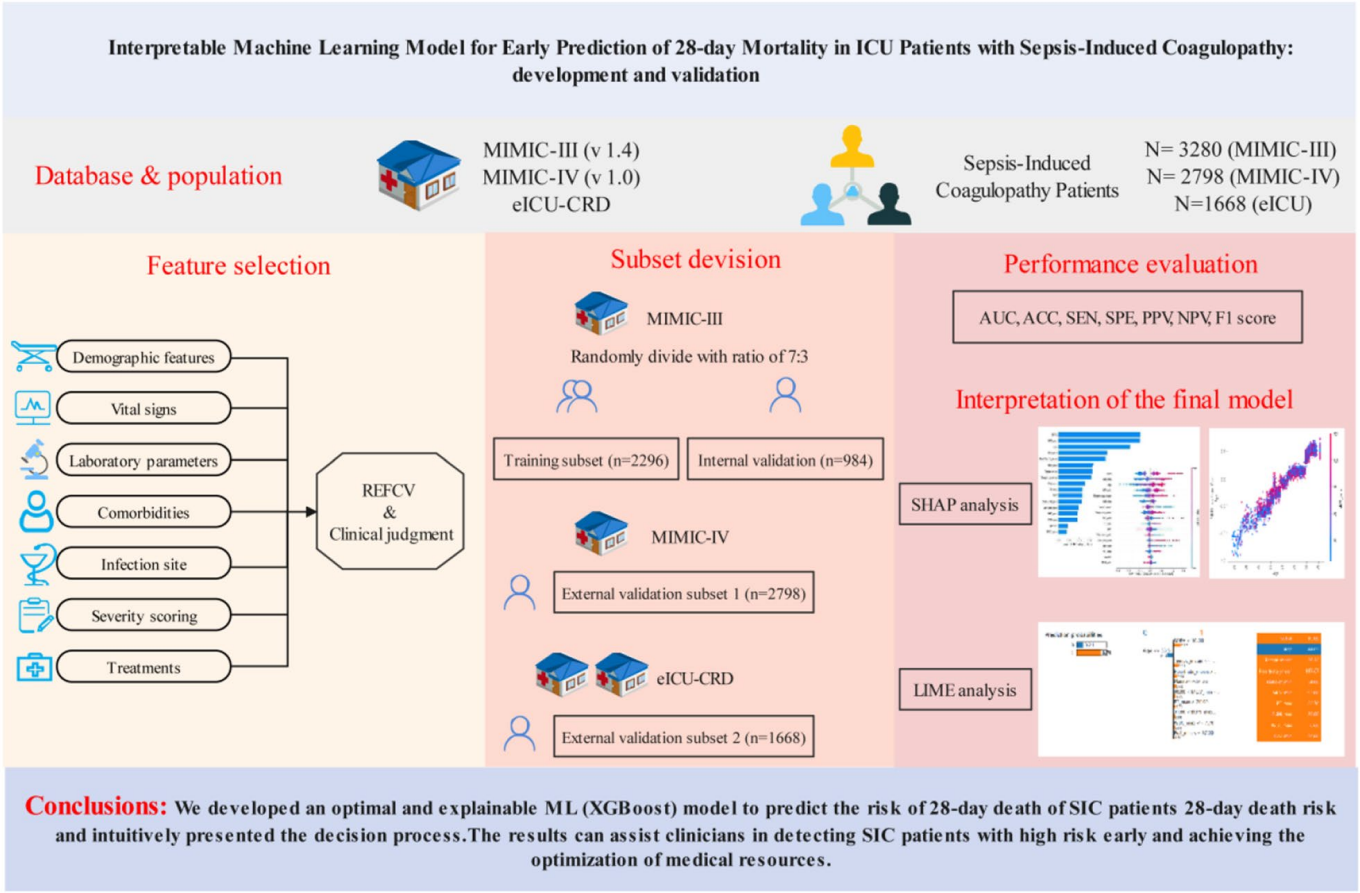

**Supplementary Information:**

The online version contains supplementary material available at 10.1186/s40001-023-01593-7.

## Introduction

Sepsis is life-threatening organ dysfunction caused by a dysregulated host response to infection [1]. The global incidence is approximately 50 million person-years, which poses severe challenges to the public health systems of the countries [2]. Although research on the pathogenesis and treatment of sepsis has been carried out for decades, no specific treatment has been found so far. Currently, the hospital mortality of adults with sepsis is about 189/100,000 person-years, while the intensive care unit (ICU) mortality is as high as over 42% [3]. It is well known that coagulation abnormalities usually occur in sepsis patients. According to the International Society On thrombosis and Haemostasis Guideline, its incidence is maintained at roughly 50–70% [4]. The primary pathogenesis of coagulopathy in patients with sepsis is exceptionally complex, including massive activation of platelets and other inflammation cells (such as neutrophils and lymphocytes) and vascular endothelial damage. These mechanisms are manifested in the body's dysregulated response to inflammation, the platelet count decrease, the coagulation reaction enhancement and the anticoagulation mechanism injury, and large immune-micro thrombus formation, which in turn affects the perfusion of organs [5, 6].

Although the definition of sepsis-induced coagulopathy (SIC) is still controversial, it is generally considered that this stage is from the initial compensated period to the decompensated disseminated intravascular coagulation period. The gold standard for SIC is still unclear, and the Toshiaki Iba scale is mainly used for diagnosis, which is comprehensively judged from three aspects: the degree of thrombocytopenia, the international normalized ratio (INR) level, and the SOFA score [7]. A single-center retrospective observational trial with large samples has reported a strong association between SIC and poor outcomes in hospitalized patients [8], and any delayed or omitted interventions may be detrimental to such patients [9]. Thus, it is of utmost importance to identify the high-risk group of SIC patients and implement timely intervention therapy, which is essential to reduce mortality. To date, no single recognized evaluation criteria have been found to predict the prognosis of patients with SIC accurately.

Therefore, this present study aimed to establish a machine learning (ML) model for early prediction of 28-day death in SIC patients based on the Medical Information Mart for Intensive Care III (MIMIC-III) database and further validated in MIMIC-IV and eICU-CRD databases. In addition, we adopted the Shapley additive explanation (SHAP) and Local Interpretable Model-Agnostic Explanations (LIME) methods to provide a reasonable interpretation for the prediction results and assisted the clinical practice of intensivists and relevant researchers.

## Methods

### Data sources

Data used in this present study were obtained from three large, open-access databases called MIMIC-III (v 1.4), MIMIC-IV (v 1.0) and eICU-CRD. The MIMIC-III (v 1.4) contained comprehensive records of 46,520 patients admitted to the Beth Israel Deaconess Medical Center in Boston, Massachusetts, between June 2001 and October 2012 [10]; while the MIMIC-IV (v 1.0) comprised almost 300,000 patients at the same center who were admitted between 2008 and 2019 [11]. Furthermore, the eICU-CRD database is a multicenter database of over 200,000 ICU admissions in the United States. Considering the partially overlapping patients in MIMIC-III and MIMIC-IV datasets, we extracted the patients from 2012 to 2019 at the MIMIC-IV set using MIMIC-III Clinical Database CareVue subset (2001–2008) and the admission time [12]. The relevant clinical data included demographic characteristics, vital signs, laboratory results, imaging examinations, microbial culture results, medication and procedures records, survival information, and a data dictionary. To achieve authorization, users must complete the collaborative institution training initiative program course by the US National Institutes of Health. Zhou and Lu have finished the online examination and obtained a certification number (Record ID: 53186220, 38455175). Since the MIMIC and eICU-CRD are both publicly available anonymized databases, approval from the ethical committee was exempted.

### Study population

Septic patients diagnosed with SIC on the first day of ICU admission were eligible for inclusion in the study. Only the first stay was included for analysis if patients were admitted to ICU more than once. The definition of sepsis was based on the Third International Consensus Definitions for Sepsis and Septic Shock (Sepsis-3), that is, patients with confirmed or suspected infection and a total SOFA score ≧ 2 [1]. Suspected infection refers to antibiotics administered within three days of the date of culture collection. According to Toshiaki Iba's rating scale, SIC was identified based on the PT-INR level, platelet count, and SOFA score [7]. The details about the SIC diagnostic criteria can be found in Additional file 3: Table S3. The exclusion criteria were (1) minors (< 18 years old); (2) pregnant women; (3) patients with congenital coagulopathy; (4) patients with neoplasm were also excluded, taking into account the effect of tumors and related chemotherapy agents on the coagulation function; (5) ICU stays less than 48 h.

### Data extraction and feature engineering

PostgreSQL programming (v 4.21) and STATA software (v 15.1) were used to extract data and concatenate each list based on the specific hadm_id or stay_id code. The following information was extracted, including age, gender, weight, ICU types, comorbidities, SOFA (which excluded the platelet), LODS, SAPS II, SIC score, vital signs, laboratory parameters, infection site, mechanical ventilation use, norepinephrine use, and survival record. The average of each vital sign within the first 24 h after ICU entry was calculated and used for the analysis, including heart rate (HR), mean blood pressure (MBP), respiratory rate (RR), and temperature. The laboratory parameter value associated with the greatest severity of illness during the first 24 h after ICU admission was extracted (except for mean blood glucose concentration), including aniongap_max, bicarbonate_min, chloride_max, hematocrit_min, hemoglobin_min, lactate_max, platelet count_min, potassium_max, partial prothrombin time_max (PTT), INR, prothrombin time_max (PT), sodium_min, blood urea nitrogen_max (BUN), white blood cells_max (WBC), PO_2_-min, PCO_2_-max, PH-min, mean corpuscular hemoglobin concentration_min (MCHC), red blood cell distribution width_max (RDW), mean corpuscular volume_min (MCV), alanine aminotransferase_max (ALT), aspartate aminotransferase_max (AST), bilirubin, creatinine_max etc.. Comorbidities were identified by the International Classification of Diseases, Ninth Revision (ICD-9), combining with Tenth Revision (ICD-10) diagnosis codes when discharge, including hypertension, chronic obstructive pulmonary disease, diabetes, myocardial infarction, chronic heart failure, and liver disease. The outcome of this present study is the 28-day mortality following ICU admission. The patient was considered a survivor if there was no record of death_time within 28-day after ICU admission.

The feature engineering was completed in three steps. Firstly, missing value identifying and processing. In this study, we used the package “VIM” to recognize the distribution of missing values. Besides, features with more than 30% missing values were removed, such as ALT, AST, and bilirubin. Additional file 4: Figure S1 shows the percentage of missing values in each database. For the remaining features, missing values were imputed using the package “randomForest” of R. Secondly, outliers identifying and processing. Within normally distributed data, outliers were identified based on the 3σ principle. Furthermore, nonparametric data were tested for outliers using the interquartile range method. All outliers were eventually winsorized using the winsor2 command in STATA software. Thirdly, feature selection for model construction. Feature selection was performed by a tenfold Recursive Feature Elimination Cross-Validation (RFECV) based on a random forest regressor in the training set. RFE, as a greedy algorithm, ranked and selected features according to their importance by iterative training [13].

### Statistical analysis

Normal distribution was assessed with Agostino tests. The continuous variables were presented as mean (standard deviation) or median (interquartile ranges, IQR) according to the type of data distribution and compared by unpaired Student's test or Mann–Whitney U-test. Categorical variables were compared using the *χ*^2^ or Fisher exact test.

**Fig. 1:**
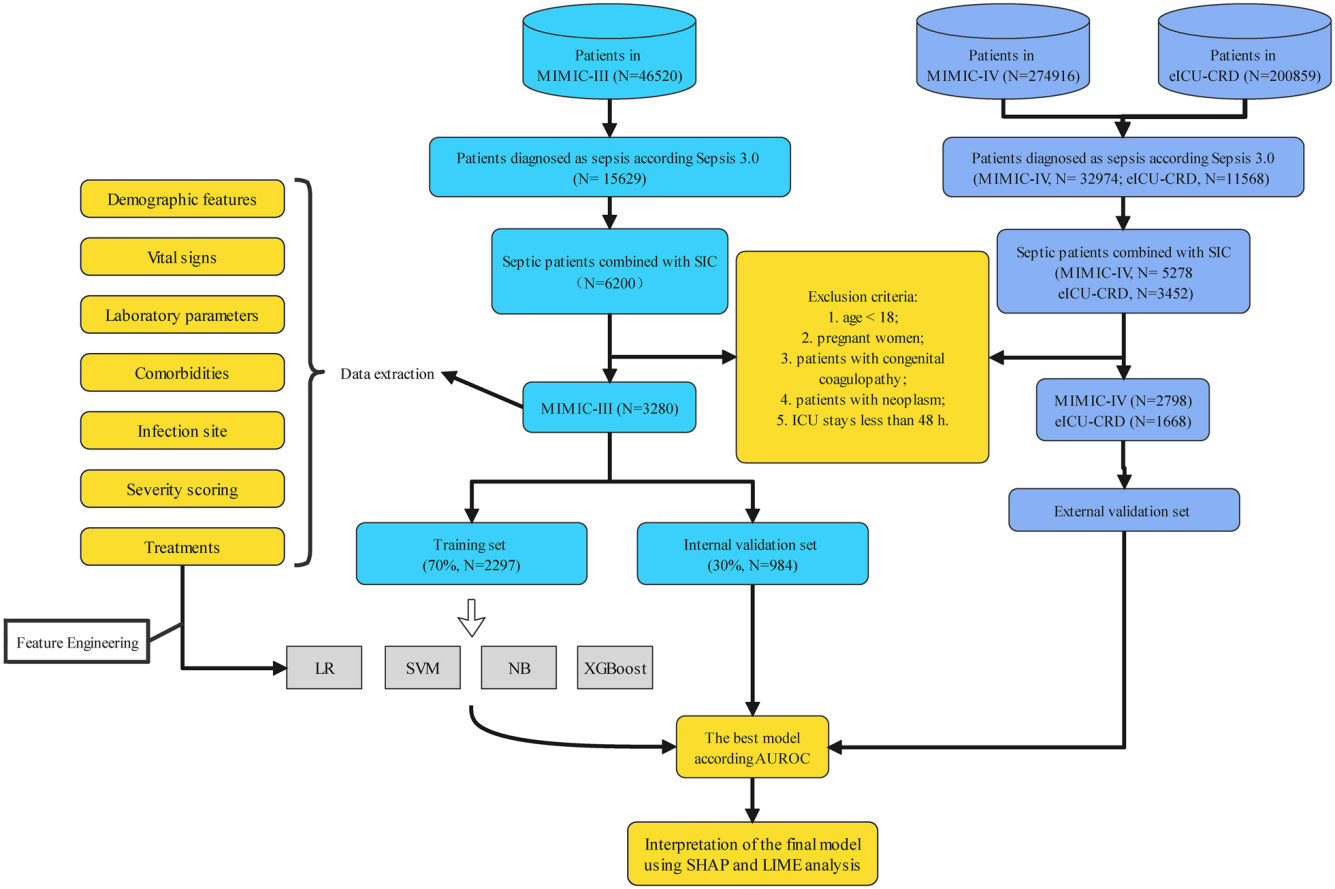
The flowchart and framework of the prediction models

The MIMIC-III database was randomly assigned with 70% for training and 30% for internal validation, while the MIMIC-IV and EICU database was used for external validation. Four machine learning methods (logistic regression-LR, XGBoost, support vector machine-SVM, and naive bayesian-NB) and three severity scoring systems (SOFA, SAPS II and SIC score) were, respectively, used to develop models for the ICU 28-day death prediction in SIC patients. We applied a tenfold cross-validated grid-search approach to the predefined models to achieve optimal parameters. The main parameters of XGBoost in this study were set as follows: n_estimators = 30, learning_rate = 0.23, max_depth = 3, gamma = 0. Areas under the receiver operating characteristic curve (AUROC), area under the precision-recall curve (AUPRC), accuracy, sensitivity, specificity, positive predictive value (PPV), negative predictive value (NPV), and F_1_ score were all calculated to evaluate the prediction performance of each model. All comparisons of AUROCs were performed as two-sided DeLong Tests. While, the F_1_ score is the harmonic mean of precision and recall, that is defined as follows: F_1_ = 2 × Precision x Recall/(Precision + Recall) [14]. SHAP and LIME algorithms were commonly applied in explaining the output of the machine learning model [15, 16]. The former applied a game-theoretic approach to evaluate feature contributions toward any model prediction and identify the features most prominently influenced by the provision of SHAP values [17]. In this study, both were used to explain the final prediction model with contributing risk factors resulting in ICU 28-day death in patients with SIC. In addition, the partial dependence plot of each feature contained in the final model was drawn using the "dependence plot" function to assess the connection between each feature and the risk of ICU 28-day death. All statistical analyses were performed using R software (v 3.6.2) and Python software (v 3.8.5). The framework of the prediction models is shown in Fig. 1.

## Results

### Baseline characteristic

After applying the inclusion and exclusion criteria, 3280 SIC patients were identified from the MIMIC-III database, while 2798 and 1668 SIC patients were from the MIMIC-IV and e-CIU database respectively. Subsequently, patients included in the MIMIC-III database were randomly assigned to training (*N* = 2296) and internal validation cohort (*N* = 984) with the ratio of 7:3. As shown in Table 1, the ICU 28 day mortality was 33.9% (779/2296) and 34.0% (335/984) in the training and internal validation cohort. SIC patients who died on the day 28 after ICU entry had an older age (68.34 and 69.28 in the training and internal validation cohort), higher proportion of liver disease (30% and 33%), and higher severity score (SOFA: 9 and 9; LODS: 8 and 8; SAPS II: 53 and 54) compared with survivors. Regarding the SIC score, the percentage of elevated SIC sore in non-survivors was significantly higher than in survivors in the training and internal validation cohort (Table 1). Meanwhile, non-survivors had faster mean HR (91.96 and 92.90 min^−1^ in training and internal validation cohort) and RR (20.6 and 20.17 min^−1^), lower MBP (72.42 and 71.63 mmHg) and temperature (36.71 and 36.72 ℃) within 24 h after ICU admission. Significant abnormalities in blood coagulation indexes, such as prolonged PTT (45 and 43.1 s in training and internal validation cohort) and PT time (18.3 and 18.9 s), higher INR (1.9 and 1.9), RDW (16.5 and 16.7%), and MCV (92 and 92.79 fL), were noted in the non-survivors group. The characteristics of the included SIC patients from the MIMIC-IV and eICU-CRD database were presented in Additional file 1: Table S1 and Additional file 2: Table S2.

**Table 1:** The comparison of baseline demographics and clinical characteristics between surviving patients and those that died in training and internal validation sets

Cohort	Training set (*n* = 2296)			Internal validation set (*n* = 984)		
Variables	Survival in ICU 28 day (*n* = 1517)	Death in ICU 28 day (*n* = 779)	*P*	Survival in ICU 28 day (*n* = 649)	Death in ICU 28 day (*n* = 335)	*P*
Gender, *n* (%)			0.28			0.642
Male	890 (59)	476 (61)		395 (61)	198 (59)	
Female	627 (41)	303 (39)		254 (39)	137 (41)	
Age (Years)	66.14 (52.95, 78.42)	68.34 (55.70, 80.27)	0.004	66.47 (51.43, 78.49)	69.28 (54.85, 80.58)	0.028
Weight (kg)	79.95 (67.78, 94)	78.2 (65.32, 92.3)	0.114	80 (67.33, 95)	76 (63.3, 91)	0.011
ICU type			< 0.001			< 0.001
CCU	136 ( 9)	83 (11)		60 (9)	36 (11)	
CSRU	246 (16)	37 (5)		96 (15)	15 (4)	
MICU	765 (50)	511 (66)		336 (52)	222 (66)	
SICU	210 (14)	95 (12)		96 (15)	37 (11)	
TSICU	160 (11)	53 (7)		61 (9)	25 (7)	
Comorbidity, *n* (%)						
Hypertension, *n* (%)	518 (34)	234 (30)	0.053	211 (33)	77 (23)	0.002
COPD, *n* (%)	32 (2)	25 (3)	0.144	8 (1)	5 (1)	0.772
Diabetes, *n* (%)	424 (28)	183 (23)	0.025	184 (28)	79 (24)	0.127
MI, *n* (%)	32 (2)	24 (3)	0.198	16 (2)	10 (3)	0.786
CHF, *n* (%)	36 (2)	24 (3)	0.385	19 (3)	12 (4)	0.716
Liver disease, *n* (%)	305 (20)	232 (30)	< 0.001	131 (20)	112 (33)	< 0.001
Severity score						
SOFA	6.00 (5.00, 9.00)	9.00 (7.00, 13.00)	< 0.001	7.00 (5.00, 9.00)	9.00 (7.00, 13.00)	< 0.001
LODS	5.00 (4.00, 7.00)	8.00 (6.00, 10.00)	< 0.001	5.00 (4.00, 7.00)	8.00 (6.00, 10.00)	< 0.001
SAPS II	40.00 (32.00, 49.00)	53.00 (45.00, 64.00)	< 0.001	40.00 (33.00, 49.00)	54.00 (43.00, 66.00)	< 0.001
SIC score, *n* (%)			< 0.001			< 0.001
4	441 (29)	129 (16)		171 (26)	54 (16)	
5	643 (42)	294 (38)		291 (45)	120 (36)	
6	429 (28)	360 (46)		191 (29)	157 (47)	
Vital signs^a^						
Mean heartrate, (min^−1^)	87.70 (77.67, 98.29)	91.65 (81.06, 106.12)	< 0.001	87.98 (78.96, 100.13)	92.90 (82.24, 106.41)	< 0.001
MAP, (mmHg)	75.23 (69.86, 81.60)	72.42 (66.33, 78.10)	< 0.001	74.33 (69.09, 80.00)	71.63 (64.35, 77.63)	< 0.001
Mean resp. rate, (min^−1^)	19.19 (16.45, 22.00)	20.60 (18.09, 24.00)	< 0.001	19.21 (16.75, 21.71)	20.17 (17.82, 24.84)	< 0.001
Mean temperature, (℃)	36.88 (36.48, 37.30)	36.71 (36.13, 37.14)	< 0.001	36.88 (36.51, 37.28)	36.72 (36.26, 37.16)	< 0.001
Laboratory tests^b^						
Mean glucose, (mg/dl)	134.00 (114.50, 156.75)	135.89 (110.71, 163.63)	0.563	136.60 (111.82, 156.32)	137.40 (109.54, 161.92)	0.99
Aniongap_max	16.00 (13.00, 19.00)	18.00 (14.88, 22.00)	< 0.001	15.00 (13.00, 18.00)	18.00 (14.50, 22.00)	< 0.001
Bicarbonate_min, (mEq/L)	21.00 (18.00, 24.00)	19.00 (15.00, 23.00)	< 0.001	21.00 (18.00, 23.00)	19.00 (15.00, 23.00)	< 0.001
Chloride_max, (mEq/L)	109.00 (105.00, 113.00)	107.00 (102.00, 113.00)	< 0.001	109.00 (105.00, 113.00)	107.00 (102.00, 113.00)	< 0.001
Hematocrit_min, (%)	27.00 (23.00, 31.50)	26.60 (23.00, 30.80)	0.277	26.60 (22.70, 31.00)	26.60 (23.30, 30.45)	0.574
Hemoglobin_min, (g/dL)	9.20 (7.90, 10.70)	9.00 (7.85, 10.40)	0.082	9.10 (7.80, 10.60)	9.20 (7.90, 10.45)	0.949
Lactate_max, (mmol/L)	2.83 (2.10, 4.20)	3.49 (2.40, 6.50)	< 0.001	2.89 (2.00, 4.40)	3.60 (2.30, 6.77)	< 0.001
Lowest platelet level, (K/uL)	100.00 (67.00, 124.00)	79.00 (46.00, 115.00)	< 0.001	99.00 (65.00, 123.00)	80.00 (47.00, 113.50)	< 0.001
Potassium_max, (K/uL)	4.60 (4.10, 5.20)	4.60 (4.10, 5.45)	0.018	4.50 (4.00, 5.20)	4.60 (4.10, 5.35)	0.164
PTT_max, (s)	38.80 (32.10, 53.70)	45.00 (35.20, 69.85)	< 0.001	40.00 (32.80, 57.30)	43.10 (33.95, 69.08)	0.006
INR_max,	1.60 (1.40, 1.97)	1.90 (1.50, 2.70)	< 0.001	1.60 (1.30, 2.00)	1.90 (1.40, 2.90)	< 0.001
PT_max, (s)	16.50 (14.70, 19.50)	18.30 (15.50, 24.20)	< 0.001	16.50 (14.90, 19.70)	18.90 (15.60, 25.00)	< 0.001
Sodium_min, (mEq/L)	136.00 (133.00, 139.00)	136.00 (132.00, 139.50)	0.077	136.00 (133.00, 139.00)	136.00 (132.00, 139.00)	0.102
BUN_max, (mg/dL)	27.00 (17.00, 45.00)	38.00 (25.00, 60.00)	< 0.001	26.00 (17.00, 42.00)	38.00 (23.50, 62.00)	< 0.001
WBC_max, (K/uL)	11.80 (8.00, 16.90)	12.40 (7.10, 18.85)	0.614	11.80 (7.80, 17.00)	11.40 (6.95, 18.10)	0.848
Po2-min, (mmHg)	91.00 (70.00, 103.49)	78.00 (62.00, 98.46)	< 0.001	93.00 (70.00, 103.84)	77.00 (60.00, 99.59)	< 0.001
Pco2-max, (mmHg)	46.00 (40.00, 50.00)	44.81 (37.00, 51.00)	0.031	46.43 (41.00, 50.00)	44.00 (37.00, 49.00)	< 0.001
PH-min	7.31 (7.26, 7.36)	7.30 (7.18, 7.37)	< 0.001	7.31 (7.26, 7.35)	7.31 (7.18, 7.35)	0.006
MCHC_min, (g/L)	33.70 (32.70, 34.70)	33.20 (32.00, 34.20)	< 0.001	33.62 ± 1.59	33.11 ± 1.57	< 0.001
RDW_max, (%)	15.00 (14.00, 16.70)	16.50 (14.84, 18.35)	< 0.001	14.90 (14.00, 16.50)	16.70 (15.00, 18.80)	< 0.001
MCV_min, (fL)	89.00 (85.00, 94.00)	92.00 (87.00, 97.00)	< 0.001	89.00 (85.00, 93.00)	92.79 (88.00, 98.00)	< 0.001
Creatinine_max, (μmol/L)	1.40 (0.90, 2.20)	1.70 (1.10, 2.90)	< 0.001	1.30 (0.90, 2.10)	1.70 (1.10, 2.90)	< 0.001
Infection site, *n* (%)						
Pulmonary infection, *n* (%)	489 (32)	283 (36)	0.055	211 (33)	102 (30)	0.558
Urinary tract, *n* (%)	458 (30)	158 (20)	< 0.001	193 (30)	75 (22)	0.017
Catheter, *n* (%)	54 ( 4)	12 (2)	0.009	14 (2)	7 (2)	1
Septicemic, *n* (%)	21 ( 1)	12 (2)	0.91	10 (2)	5 (1)	1
Treatment measures, *n* (%)						
MV, *n* (%)	373 (25)	222 (28)	0.048	168 (26)	91 (27)	0.723
Norepinephrine, *n* (%)	279 (18)	296 (38)	< 0.001	143 (22)	139 (41)	< 0.001

Categorical data were presented as frequency (percentage), parametric continuous data were presented as mean ± (standard deviation), whereas non-parametric continuous data were presented as median (interquartile ranges)

*COPD* Chronic Obstructive Pulmonary Disease, *MI* Myocardial Infarction, *CHF* Chronic Heart Failure, *SOFA* Sequential Organ Failure Assessment, *LODS* Logistic Organ Dysfunction System, *SAPS II* Simplified acute physiology II, *PT* Prothrombin Time, *PTT* Partial Thromboplastin Time, *INR* International Normalized Ratio, *BUN* Blood Urea Nitrogen, *MCHC* Mean Corpuscular Hemoglobin Concentration, *RDW* Red Blood Cell Distribution Widths, *MCV* Mean Corpuscular Volume, *MV* Mechanical Ventilation, *MAP* Mean arterial pressure

^a^Vital signs were calculated as mean value during the first 24 h since ICU admission of each included patients

^b^The laboratory tests recorded the worst value during the first 24 h since ICU admission of each included patients

### Prediction model building and evaluation

Before prediction model construction, 20 features were preliminarily screened out using RFECV, including age, SOFA score, LODS score, HR_mean, systolic pressure_mean, MBP, RR_mean, temperature_min, lactate_max, platelet count_min, PTT_max, PT_max, INR_max, BUN_max, WBC_max count, PaO2_min, PH_min, MCHC_min, RDW_max, and MCV_min. However, systolic pressure, INR, PH, and LODS were deleted from the original features list, and gender was included based on expert consultations and clinical judgment results. Thus, 17 features were eventually included for further model building. Additional file 6: Figure S3 presents how the accuracy varies with the number of features in RFECV processing. Considering the potential bias results from the discrepancy of missing values, we further analyzed the differences between missing values for each of the 17 features between survivors and non-survivors in three databases. Moreover, Additional file 5: Figure S2 indicates that there was no significantly different in the distribution of the missing values between survivors and non-survivors in each database.

**Fig. 2:**
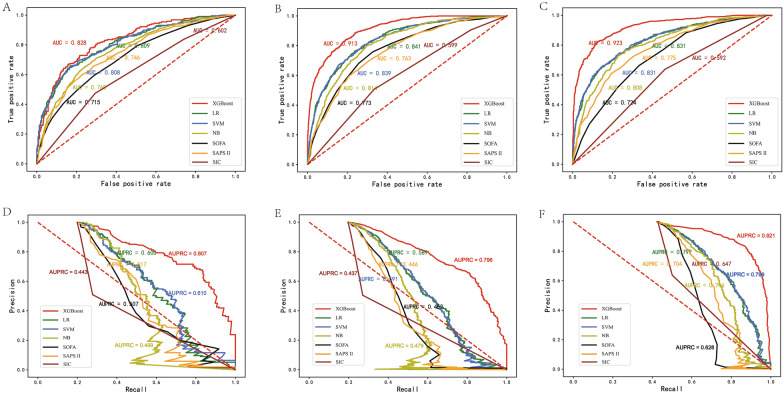
Receiver operating characteristic curves and area under the precision recall curve showing 28-day death of SIC patients predictive performance of two severity scoring and four machine learning algorithms based on the selected features in the internal validation set (MIMIC-III) (**A**, **D**), MIMIC-IV (**B**, **E**), and eICU-CRD (**C**, **F**) database. *LR* logistic regression, *NG* naive bayes, *SVM* support vector machine, *SOFA* sequential organ failure assessment, *SAPS II* simplified acute physiology score II, *SIC* sepsis-induced coagulopathy, *AUC* area under the receiver operating characteristic curve

We utilized four machine learning models, XGBoost, LR, SVM, and NB, with these 17 features mentioned above to predict the risk of ICU 28-day death in SIC patients. The results demonstrated that the XGBoost presented the largest AUROC compare with other models in internal validation cohort and external validation cohort [internal validation cohort: 0.828 95% confidence interval (CI) 0.795, 0.861; MIMIC-IV: 0.913 95% CI 0.905, 0.932; eICU-CRD: 0.923 95% CI 0.913, 0.941] (Fig. 2A–C and Table 2), and these differences were significant when compared by DeLong test (*P* < 0.001). However, the results of AUROC may be insensitive due to the imbalance distribution of data; hence, we analyzed the AUPRC value of each model. The results presented that the XGBoost also performed best in three cohorts [internal validation cohort: 0.807 95% confidence interval (CI) 0.743, 0.864; MIMIC-IV: 0.796 95% CI 0.703, 0.884; eICU-CRD: 0.921 95% CI 0.874, 0.955] (Fig. 2D, E and Table 2). Besides, XGBoost outperformed the other algorithms, SOFA, SAPS II and SIC score on the aspect of accuracy (internal validation cohort: 0.785; MIMIC-IV cohort: 0.885; EICU cohort: 0.891) and F1score (internal validation cohort:0.63; MIMIC-IV cohort: 0.69; EICU cohort: 0.70). In addition, we drew the calibration plots using the bootstrap method and performed the decision curve analysis (DCA) of each model in three databases. As shown in Additional file 6: Figure S3D, E, F, the bias-corrected line slightly deviated from the ideal line, indicating a good agreement between the prediction and observation. And the DCA results demonstrated that the XGBoost model provided a greater net benefit when the threshold probability was within 0 and 1 in both databases (Additional file 6: Figure S3A, B, C). Therefore, we selected the XGBoost for all further analyses.

**Table 2:** The prediction performance of each model in internal validation and external validation sets

Model	AUROC	AUPRC	ACC	SE	SP	PPV	NPV	F1 score	DeLong test for AUC
Internal validation set									
XGBoost	0.828	0.807	0.785	0.646	0.904	0.739	0.2	0.63	Reference
LR	0.809	0.605	0.770	0.546	0.886	0.712	0.209	0.62	*P* < 0.001^*^
NB	0.768	0.499	0.733	0.481	0.862	0.643	0.236	0.55	*P* < 0.001^*^
SVM	0.808	0.610	0.774	0.504	0.914	0.751	0.219	0.60	*P* < 0.001^*^
SOFA	0.715	0.507	0.713	0.310	0.921	0.671	0.279	0.42	*P* < 0.001^*^
SAPS II	0.746	0.517	0.722	0.397	0.889	0.649	0.259	0.49	*P* < 0.001^*^
SIC score	0.602	0.443	0.664	0.212	0.964	0.431	0.336	0.35	*P* < 0.001^*^
External validation set (MIMIC-IV)									
XGBoost	0.913	0.796	0.885	0.523	0.974	0.828	0.107	0.69	Reference
LR	0.841	0.589	0.847	0.403	0.956	0.692	0.133	0.58	*P* < 0.001^*^
NB	0.814	0.479	0.807	0.511	0.879	0.510	0.200	0.51	*P* < 0.001^*^
SVM	0.839	0.591	0.842	0.347	0.964	0.700	0.142	0.54	*P* < 0.001^*^
SOFA	0.773	0.452	0.713	0.310	0.921	0.671	0.279	0.48	*P* < 0.001^*^
SAPS II	0.763	0.446	0.816	0.192	0.970	0.606	0.170	0.47	*P* < 0.001^*^
SIC score	0.599	0.437	0.803	0.121	0.982	0.478	0.197	0.35	*P* < 0.001^*^
External validation set (EICU)									
XGBoost	0.923	0.921	0.891	0.555	0.976	0.842	0.102	0.70	Reference
LR	0.831	0.797	0.847	0.403	0.956	0.692	0.133	0.58	*P* < 0.001^*^
NB	0.808	0.744	0.744	0.628	0.829	0.728	0.247	0.50	*P* < 0.001^*^
SVM	0.831	0.799	0.770	0.645	0.861	0.772	0.231	0.58	*P* < 0.001^*^
SOFA	0.724	0.628	0.665	0.518	0.772	0.624	0.313	0.43	*P* < 0.001^*^
SAPS II	0.775	0.704	0.718	0.518	0.772	0.624	0.313	0.48	*P* < 0.001^*^
SIC score	0.592	0.647	0.579	0.638	0.536	0.501	0.330	0.35	*P* < 0.001^*^

*LR* regression model, *NB* naive bayes, *SVM* support vector machine, *SOFA* sequential organ failure assessment, *SAPS II* simplified acute physiology score II, *AUROC* Area Under the Receiver Operating Characteristic Curve, *AUPRC* Area Under the Precision-Recall Curve

* indicates a statistical difference

### Explanation of risk factors

**Fig. 3:**
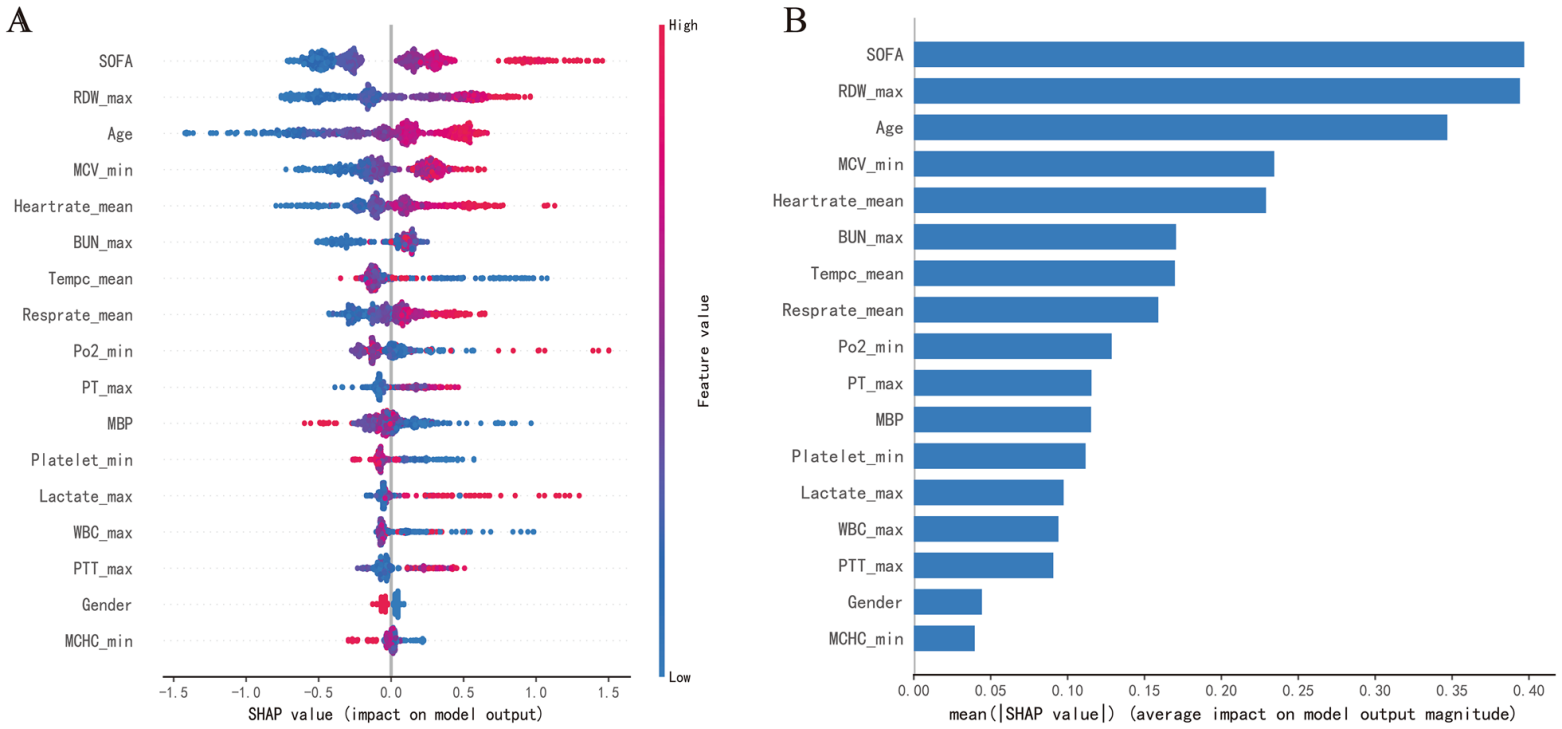
The interpretation of the XGBoost model. **A** Feature importance ranking based on SHAP values. The position on the Y-axis implied the importance ranking, and the X-axis reflected the association between each value of features and the corresponding SHAP value. **B** The importance ranking of included features according to the mean (|SHAP value|). *SOFA* sequential organ failure assessment, *RDW* red blood cell distribution width, *MCV* mean corpuscular volume, *BUN* blood urea nitrogen, *MBP* mean blood pressure, *WBC* white blood cell, *MCHC* mean corpuscular hemoglobin concentration

The importance score of 17 features used in the XGBoost model has been calculated to identify the critical features (Fig. 3A). The position on the Y-axis implied the importance ranking, and the X-axis reflected the association between each value of features and the corresponding SHAP value. For instance, the SHAP values for advanced age are generally greater than zero, indicating that with increasing age, the risk of death also increased in SIC patients. In addition, Fig. 3B displays the ranking of the features based on the average absolute SHAP value. The permutation importance results indicated that the top five risk features were SOFA score, RDW-max, age, MCV-min, and mean HR.

**Fig. 4:**
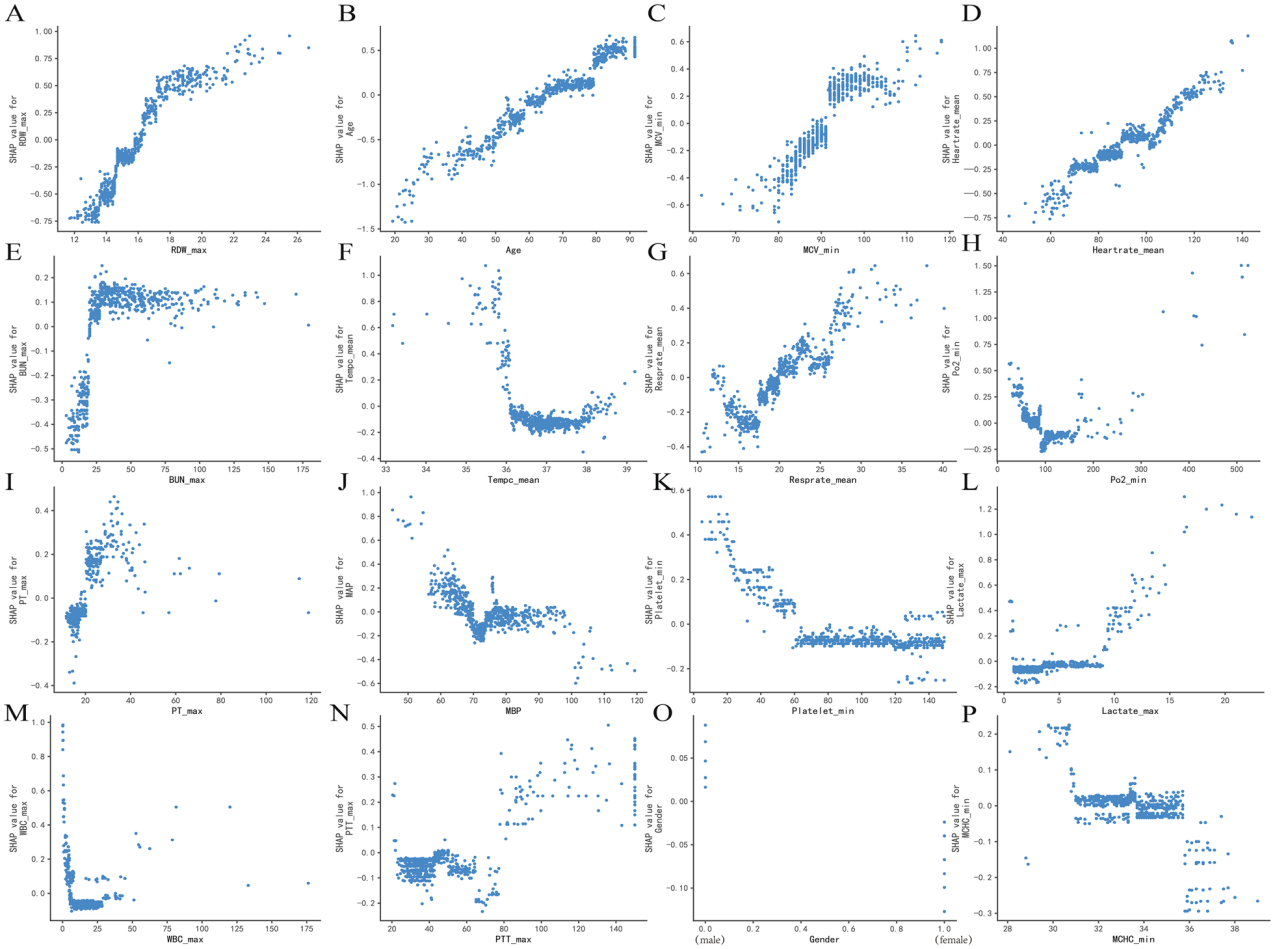
The partial dependence plots of the XGboost model based on SHAP. A-P show how the RDW_max, age, MCV_min, Heartrate_mean, Tempc_mean, Resprate_mean, Po2_min, PT_max, MAP, platelet_min, lactate_max, WBC_max, PTT_max, gender and MCHC_min affects the output of the XGBoost prediction model respectively. As the SHAP value exceeds zero, it indicated a promoting effect on the 28-day death risk. RDW=red blood cell distribution width; MCV=mean corpuscular volume; BUN=blood urea nitrogen; MBP=mean blood pressure; WBC=white blood cell; MCHC=mean corpuscular hemoglobin concentration

The partial dependence plot results showed the effect of a single feature on the output of the XGBoost model. As the SHAP value exceeds zero, it indicated a promoting effect on the outcome (Fig. 4). This study found a positive but no linear association between RDW-max, age, MCV-min, mean HR, mean RR, PT-max and death risk. Moreover, the risk elevated rapidly when BUN-max was above 24 mg/dL, lactate-max was above seven mmol/L, the mean temperature was below 36 ℃, PO_2_ was below 80 mmHg, MBP was below 70 mmHg, the minimum count of platelet was below 60 × 10^9^/L, and MCHC-min was below 310 g/L in the first 24 h after ICU admission. In gender, men were generally at higher risk for ICU 28-day death than women.

Furthermore, this study assessed the potential interactions between RDW-max and initial SOFA or age. As shown in Additional file 8: Figure S5A, the risk of 28-day death of SIC patients increased when their initial SOFA score was elevated. Patients with a higher level of RDW-max had a lower risk of 28-day death when SOFA ≦ 7, yet, for patients with a SOFA ≧ 8, a higher level of RDW-max appeared to provide more risk of death. In addition, the impact of RDW-max also appeared to vary with age (Additional file 8: Figure S5B). Even though an increased initial RDW-max value induced a higher risk of 28-day death for SIC patients with age ≦ 60, a trend in the opposite direction was seen when age was greater than 60.

### Interpretation of individual prediction

This present study explained the XGBoost prediction results of individual SIC patients using the SHAP and LIME, respectively. Additional file 9: Figure S6 provides two typical examples to illustrate the interpretability of SHAP. The features marked blue decreased the risk of death, while red features promoted death. Patient No.1, who belonged to the "true negative" group, was correctly predicted as a survivor (Additional file 9: Figure S6A). Patient No.2, who belonged to the "true positive" group, was correctly predicted as a non-survivor (Additional file 9: Figure S6B). The survivor was predicted to be alive due to higher mean HR (65.35 min^−1^), RDW (14.1%), BUN (16 mg/dL), platelet count (122 × 10^9^/L), MCV (89 fL), PT(14.8 s), and mean RR (19.45 min^−1^). The non-survivor was predicted to die due to elevated initial SOFA score (15), mean HR (117.7 min^−1^), RDW (17%), PT (22.7 s), mean RR (22.12 min^−1^), and decreased platelet count (34 × 10^9^/L). Besides, we conducted explanations for the two cases mentioned above based on the LIME (Additional file 10: Figure S7). The blue box indicated that the features are risk factors for ICU 28-day death, while the orange box indicated that the features are protective factors. And the LIME was similar to SHAP results.

## Discussion

In this study, we have developed and validated machine learning models using 17 selected features, including age, gender, maximum SOFA score, mean HR, MBP, mean RR, temperature, lactate-max, minimum platelet count, PTT-max, PT-max, BUN-max, maximum WBC count, PO_2_-min, MCHC-min, RDW-max, and MCV-min to predict the ICU 28-day death risk. The above features could be easily collected within 24 h after ICU admission. The performance of each model was evaluated by AUROC, accuracy, sensitivity, specificity, PPV, NPV, and F_1_ score. XGBoost achieved the best prediction results, while the RF model performed worst. In addition, the SHAP function was used to interpret the prediction results of XGBoost to help intensivists better understand the process of this model decision and provide the basis for early interventions in SIC patients with a high risk of death.

Nowadays, ML has played a crucial role in the early warning and prognosis prediction of diseases [15, 16, 18]. These algorithms can analyze complex and non-linear data and even make a real-time prediction based on time series, which cannot be completed by traditional regression analysis. However, with the continuous development of algorithms, models become increasingly complex, increasing the difficulty of interpretation. This phenomenon is often referred to as the "black box" which is not conducive to the promotion of ML in the medical and health field [19]. To illustrate how these included features affect the 28-day mortality of SIC patients, we employ the SHAP value to analyze each feature. The SHAP is different from traditional feature importance. The latter only reports the importance permutation of features but cannot identify how each feature affects the model prediction results. In comparison, the most significant advantage of SHAP is that it can reflect permutation of importance and illustrate the positive and negative effects of included features. Figure 3 showesthat the five most important factors include the patient's initial SOFA score, RDW value, age, MCV, and mean heart rate, affecting SIC patients' ICU survival for 28 days. Meanwhile, when the values of each feature are different, the impacts on the prognosis are different. In addition, the relationship between some special continuous variables and the risk of unfavorable outcomes may not always be linear. Thus, exploring these features' risk threshold or trigger point in clinical practice has become even more critical. Unfortunately, it was considerably tricky for traditional linear models, such as logistic regression or Cox regression, to accomplish this goal.

In this developed XGBoost model, we constructed each feature's partial dependence plot to analyze further the correlation between each variable and the 28-day death risk. The stability of the urea generation rate is lower than that of serum creatinine and is susceptible to factors other than the kidney. BUN significantly increases when the glomerular filtration rate is reduced by 50% [20]. Meanwhile, Gaudry et al. demonstrated that a BUN level higher than 112 mg/dL is one of the major criteria for initiating restrictive renal replacement therapy [21]. In contrast, we found that the initial BUN level has little impact on the 28-day mortality of SIC patients, and it only exhibits harmful effects when the level is greater than 24 mg/dL. SOFA score and platelet count were included in Toshiaki Iba's scale. A single-center retrospective study by Lyons PG et al. referred to the Toshiaki Iba scale classified SIC patients into three levels, and their results showed that the severity of SIC was positively correlated with the patient's hospital mortality [8]. However, in this present study, Additional file 8: Figure S5 and Additional file 7: Figure S4K, respectively, presented that the significant contribution to mortality was not observed until the initial SOFA score was higher than eight or the platelet count was lower than 60 K/uL. After that, the risk of 28-day death rapidly elevated as the SOFA score increased and platelet count decreased. Thus, it appeared that the initial SIC score is not an ideal indicator for predicting the 28-day death risk in SIC patients. Serum lactate was a common biomarker to achieve risk stratification in sepsis patients. Mikkelsen et al. categorized initial venous lactate of sepsis patients as mild (≦2 mmol/L), middle (2–3.9 mmol/L), or severe degree (≧ 4 mmol/L), and proved that middle and severe lactate degree were all significantly associated with the 28-day mortality of sepsis patients whenever the presence (aOR5.14, 95% CI 1.74–15.18, *p* = 0.003) or absence ( aOR 3.33, 95% CI 1.47–7.56, *p* = 0.004) of septic shock using multivariable logistic regression [22]. Nevertheless, Fig. 4L shows that the SHAP value of the majority of samples remains at approximately zero when lactate level is below seven mmol/L and increases rapidly when lactate level is over 7 mmol/L, except for a few deviation samples. The discrepancy maybe since the status hyperlactatemia was also affected by lactate clearance. A series of studies have confirmed strong correlations between lactate clearance and prognosis in septic shock patients, even though increased lactate concentration may indirectly suggest tissue hypoxia [23, 24]. However, ongoing hyperlactatemia or a significant increase in lactate levels may reflect the decreased clearance rather than an increased production in lactate metabolism [25]. This is typically seen in sepsis patients combined with liver dysfunction.

RDW level reflects the size heterogeneity of the erythrocytes and indicates the body's response to oxidative stress and inflammation [26]. In recent years, a growing number of researches have shown the potential value of RDW in predicting the prognosis of sepsis [27–30]. A meta-analysis that included 11 studies showed that elevated RDW was positively associated with mortality of sepsis patients (HR 1.14, 95% CI 1.09–1.20, *p* < 0.001). Besides, the related subgroup and sensitivity analysis results based on quality, infection sites, and complications also supported this view [27]. However, few studies had assessed the connection between RDW level and adverse prognosis of sepsis patients in different severity and age. As shown in Additional file 10: Figure S7, we noted that the elderly patients (age greater than 60) with a higher RDW seemed to have a lower 28-day death risk. This phenomenon was opposite to Wang et al.'s experimental observation in which a total of 117 sepsis patients were included. They found that the in-hospital mortality increased 1.18 fold for each 1% increase in RDW [31]. This difference can result from baseline discordance. In addition, the physiologic rising of RDW may occur in some unique elderly patients [32]. Overall, the effect of increased RDW on elderly patients with sepsis is still controversial, which is worthy of follow-up studies.

### Strengths and limitations

Compared with our previous study, this research project has several notable strengths. Firstly, the XGBoost has a good nonlinear fitting ability and improves prediction accuracy. Secondly, the SHAP and LIME solve the "black box" problem well for ML models. Thirdly, based on the SHAP values, we ranked the risk factors and illustrated their positive and negative effects on the 28-day mortality of SIC patients. Finally, and most importantly, we explored these features' risk threshold or trigger point based on a partial dependence plot.

However, there were some potential limitations to this study. First, we may have missed the inclusion of the sickest patients because those who died within the first 48 h of ICU admission were excluded. This implied that there are likely to be significant differences in baseline variables between patients who were included and those who were not included. Second, the specificities of the XGBoost model were 0.904 and 0.974, respectively, in the internal validation and external validation set; in contrast, the sensitivities were only 0.646 and 0.523. It suggested the presence of a sizeable false-negative rate in the prediction of 28-day mortality in SIC patients; thus, further clinical experience and medical judgment should be recommended for those where the model yield negative results. Third, despite extensive data, we could not obtain key coagulation indexes, such as D2 polymers, fibrinogen, and thrombin-anti-thrombin III complexes. This study is only the first step to building a death risk prediction model for SIC patients. In future studies, clinical ML models need to account for different domains (e.g., immunology, pathogenesis, and clinical phenotype) to identify SIC patients' progressive trajectories and develop a more accurate and reliable prediction model.

## Conclusion

In summary, our study developed an ML model based on MIMIC-III and MIMIC-IV databases to predict the risk of 28-day death of SIC patients early. The XGBoost performed better than LR, NB, SVM, SOFA, and SAPS II scores. SHAP and LIME are reliable methods to intuitively identify the related risk factors that affected the model making final predictions. The results can assist clinicians in screening SIC patients at high risk of 28-day death, contributing to the optimization of medical resources.

### Supplementary Information

**Additional file 1: Table S1**. The comparison of baseline demographics and clinical characteristics between surviving patients and those that died in the MIMIC-IV database.

**Additional file 2: Table S2**. The comparison of baseline demographics and clinical characteristics between surviving patients and those that died in the eICU-CRD database.

**Additional file 3: Table S3**. The details about the SIC diagnostic criteria.

**Additional file 4: Figure S1.** The percentage of missing values in MIMIC-III (A), MIMIC-IV (B), and eICU-CRD (C) database.

**Additional file 5: Figure S2**. The detailed comparison of the percentage between missing values for each of the 17 factors between survivors and non-survivors in MIMIC-III (A), MIMIC-IV (B), and eICU-CRD (C) database.

**Additional file 6: Figure S3**. Decision curve analysis of the XGBoost, LR, SVM, NB, SOFA, SAPS II and SIC in the internal validation set (MIMIC-III, A), MIMIC-IV (B) and eICU-CRD (C) database; calibration curves of each model in the internal validation set (MIMIC-III, D), MIMIC-IV (E) and eICU-CRD (F) database.

**Additional file 7: Figure S4**. Feature selection accuracy curve using recursive feature elimination cross-validation. The accuracy get the highest accuracy when the number of variables was 20 (represented as a solid point).

**Additional file 8: Figure S5**. The potential interactions between RDW with initial SOFA (A) and age (B). The Y-axis on the left represents the SHAP value of SOFA or age, while Y-axis on the right shows the different values of RDW. Despite SOFA or age being identical, the SHAP value corresponding to different RDW levels may be discrepancies. SOFA = sequential organ failure assessment; RDW = red blood cell distribution width.

**Additional file 9: Figure S6**. The interpretation of model prediction results with two actual samples using the SHAP. Patient No.1, who belonged to the "true negative" group, was correctly predicted as a survivor by XGBoost. Patient No.2, who belonged to the "true positive" group, was correctly predicted as a non-survivor. This plot shows significant features contributing to pushing the model output. The blue features decrease the risk of death, while red features promote death.

**Additional file 10: Figure S7.** The interpretation of model prediction results with two actual samples using the LIME. The blue box indicated that the features are risk factors for 28-day death, while the orange box suggests the features are protective factors.

## Data Availability

The datasets are available in the PhysioNet (https://physionet.org/content/mimiciv/1.0/; https://physionet.org/content/eicu-crd/2.0/; https://physionet.org/content/mimic3-carevue/1.4/; https://physionet.org/content/mimiciii/1.4/).

## Abbreviations

ML: Machine learning
SIC: Sepsis induced coagulopathy
MIMIC: Medical information mart for intensive care
RFE: Recursive feature elimination
AUROC: Area under the receiver operating characteristic curve
SHAP: Shapley additive explanations
LIME: Local interpretable model-agnostic explanations
SOFA: Sequential organ failure assessment
ICD: International classification of diseases
SAPS II: Simplified acute physiology score II
