# Supplementary material for: Interpretable machine learning model for early prediction of 28-day mortality in ICU patients with sepsis-induced coagulopathy: development and validation
Source: Eur J Med Res. 2024 Jan 3;29:14. doi: 10.1186/s40001-023-01593-7 (PMC10763177; doi:10.1186/s40001-023-01593-7)
Supplement: Supplementary file 1 — Additional file 1: Table S1. The comparison of baseline demographics and clinical characteristics between surviving patients and those that died in the MIMIC-IV database. [file 40001_2023_1593_MOESM1_ESM.doc]

Supplementary Table 1 The comparison of baseline demographics and clinical characteristics between surviving patients and those that died in the external validation set.

| **Validation setting（MIMIC-IV, n=2798）** | | | | |
| --- | --- | --- | --- | --- |
| **Variables** | **All patients** | **Survival in ICU 28 day（n=2247）** | **Death in ICU 28 day（n=551）** | ***P*** |
| **Gender, n (%)** |  |  |  | 0.109 |
| Male | 1726 (62) | 1403 (62) | 323 (59) |  |
| Female | 1072 (38) | 844 (38) | 228 (41) |  |
| **Age (Years)** | 65.95 (54.96, 77.77) | 65.82 (54.91, 77.33) | 66.47 (55.21, 80.14) | 0.093 |
| **Weight（kg）** | 80 (67.6, 94.69) | 80.2 (68, 94.7) | 78.3 (64.75, 94.6) | 0.046 |
| **ICU type** |  |  |  | <0.001 |
| CCU | 137 (5) | 105 (5) | 32 (6) |  |
| CVICU | 557 (20) | 540 (24) | 17 (3) |  |
| MICU | 903 (32) | 655 (29) | 248 (45) |  |
| MICU/SICU | 611 (22) | 471 (21) | 140 (25) |  |
| NICU | 32 (1) | 22 (1) | 10 (2) |  |
| SICU | 375 (13) | 317 (14) | 58 (11) |  |
| TSICU | 183 (7) | 137 (6) | 46 (8) |  |
| **Comorbidity, n(%)** |  |  |  |  |
| Hypertension, n(%) | 572 (20) | 475 (21) | 97 (18) | 0.074 |
| COPD, n(%) | 628 (22) | 492 (22) | 136 (25) | 0.178 |
| Diabetes, n(%) | 787 (28) | 634 (28) | 153 (28) | 0.876 |
| MI, n(%) | 394 (14) | 298 (13) | 96 (17) | 0.014 |
| CHF, n(%) | 769 (27) | 628 (28) | 141 (26) | 0.29 |
| Liver disease, n (%) | 1052 (38) | 788 (35) | 264 (48) | < 0.001 |
| **Severity Score** |  |  |  |  |
| SOFA | 8 (6, 12) | 8 (6, 11) | 12 (10, 15) | < 0.001 |
| LODS | 6 (4, 9) | 6 (4, 8) | 10 (8, 13) | < 0.001 |
| SAPS II | 42 (33, 52) | 39 (31, 49) | 54 (44, 65) | < 0.001 |
| SIC score, n (%) |  |  |  | < 0.001 |
| 4 | 458 (16) | 406 (18) | 52 (9) |  |
| 5 | 1315 (47) | 1094 (49) | 221 (40) |  |
| 6 | 1025 (37) | 747 (33) | 278 (50) |  |
| **Vital signs**^a^ |  |  |  |  |
| Mean heartrate, (min^−1^) | 88.39 (77.42, 100.37) | 86.89 (76.48, 98.48) | 94.97 (82.79, 106.91) | < 0.001 |
| MAP, (mmHg) | 73.23 (68.09, 79.15) | 73.69 (68.89, 79.52) | 71.26 (65.61, 77.14) | < 0.001 |
| Mean resprate, (min^−1^) | 19.15 (16.8, 22.58) | 18.76 (16.55, 21.81) | 21.38 (18.53, 24.93) | < 0.001 |
| Mean temperature, (℃) | 36.83 (36.58, 37.16) | 36.84 (36.62, 37.18) | 36.72 (36.38, 37.02) | < 0.001 |
| **Laboratory tests**^b^ |  |  |  |  |
| Mean glucose, (mg/dl) | 131. 50 (113, 156.17) | 127.20 (112.00, 153.30) | 139.32 (118.27, 164.89) | < 0.001 |
| Aniongap_max | 17 (14, 21) | 16 (13, 20) | 20 (17, 25) | < 0.001 |
| Bicarbonate_min, (mEq/L) | 20 (16, 22) | 20 (17, 23) | 17 (12, 21) | < 0.001 |
| Chloride_max, (mEq/L) | 107 (103, 111) | 107 (103, 111) | 106 (101, 111) | < 0.001 |
| Hematocrit_min, (%) | 26.5 (22.6, 31.3) | 26.6 (22.8, 31.4) | 25.7 (21.6, 31) | 0.009 |
| Hemoglobin_min, (g/dL) | 8.8 (7.5, 10.4) | 9 (7.6, 10.4) | 8.5 (7.1, 10.3) | < 0.001 |
| Lactate_max, (mmol/L) | 3 (2.3, 4.5) | 2.93 (2.2, 4) | 4.1 (2.7, 7.29) | < 0.001 |
| Lowest platelet level, (K/uL) | 86 (55, 112) | 89 (60, 114) | 68 (41, 105) | < 0.001 |
| Potassium_max, (K/uL) | 4.5 (4.1, 5.1) | 4.5 (4.1, 5) | 4.8 (4.2, 5.6) | < 0.001 |
| PTT_max, (s) | 39.4 (32.82, 56.6) | 38.2 (32.3, 52) | 49 (36, 71.9) | < 0.001 |
| INR_max, | 1.7 (1.5, 2.3) | 1.7 (1.5, 2.1) | 2.2 (1.7, 3.2) | < 0.001 |
| PT_max, (s) | 19 (16.6, 24.8) | 18.4 (16.5, 23) | 23 (18, 33.8) | < 0.001 |
| Sodium_min, (mEq/L) | 136 (133, 139) | 136 (134, 139) | 135 (131, 139) | < 0.001 |
| BUN_max, (mg/dL) | 28 (17, 46) | 25 (16, 41) | 43 (26, 64.5) | < 0.001 |
| WBC_max, (K/uL) | 13.2 (8.6, 19) | 13.1 (8.6, 18.7) | 13.5 (8.15, 20.55) | 0.499 |
| Po2-min, (mmHg) | 69 (41, 98.13) | 77 (43, 100.49) | 49 (37, 75) | < 0.001 |
| Pco2-max, (mmHg) | 45.86 (40, 50) | 45.95 (40, 50) | 45.38 (38, 54) | 0.884 |
| PH-min | 7.32 (7.25, 7.35) | 7.32 (7.26, 7.35) | 7.27 (7.15, 7.34) | < 0.001 |
| MCHC_min, (g/L) | 32.6 (31.4, 33.6) | 32.7 (31.6, 33.7) | 32 (30.9, 33.3) | < 0.001 |
| RDW_max, (%) | 15.8 (14.3, 17.9) | 15.5 (14.1, 17.4) | 17.2 (15.3, 19.9) | < 0.001 |
| MCV_min, (fL) | 91 (87, 96) | 91 (86, 96) | 93 (88, 100) | < 0.001 |
| Creatinine_max, (μmol/L) | 1.4 (0.9, 2.2) | 1.2 (0.9, 2) | 2 (1.3, 3) | < 0.001 |
| **Infection site, n(%)** |  |  |  |  |
| Pulmonary infection, n(%) | 704 (25) | 513 (23) | 191 (35) | < 0.001 |
| Urinary tract, n(%) | 436 (16) | 357 (16) | 79 (14) | 0.514 |
| Catheter, n(%) | 74 (3) | 64 (3) | 10 (2) | 0.385 |
| Septicemic, n(%) | 1171 (42) | 827 (37) | 344 (62) | < 0.001 |
| **Treatment measures, n(%)** |  |  |  |  |
| MV, n(%) | 1490 (53) | 1114 (50) | 376 (68) | < 0.001 |
| Norepinephrine, n(%) | 1107 (40) | 790 (35) | 317 (58) | < 0.001 |

Categorical data were presented as frequency (percentage), parametric continuous data were presented as mean ± (standard deviation), whereas non-parametric continuous data were presented as median (interquartile ranges);

^a^Vital signs were calculated as mean value during the first 24 h since ICU admission of each included patients;

^b^The laboratory tests recorded the worest value during the first 24 h since ICU admission of each included patients;

COPD Chronic Obstructive Pulmoriary Disease, MI Myocardial Infarction, CHF Chronic Heart Failure, SOFA Sequential Organ Failure Assessment, LODS Logistic Organ Dysfunction System, SAPS II Simplified acute physiology II, PT Prothrombin Time, PTT Partial Thromboplastin Time, INR International Normalized Ratio, BUN Blood Urea Nitrogen, MCHC Mean Corpuscular Hemoglobin Contentration, RDW Red Blood Cell Distribution Widths, MCV Mean Corpuscular Volume, MV Mechanical Ventilation, MAP Mean arterial pressure
