# Supplementary material for: Interpretable machine learning model for early prediction of 28-day mortality in ICU patients with sepsis-induced coagulopathy: development and validation
Source: Eur J Med Res. 2024 Jan 3;29:14. doi: 10.1186/s40001-023-01593-7 (PMC10763177; doi:10.1186/s40001-023-01593-7)
Supplement: Supplementary file 3 — Additional file 3: Table S3. The details about the SIC diagnostic criteria. [file 40001_2023_1593_MOESM3_ESM.doc]

| Scoring for the diagnosis of sepsis-induced coagulopathy | | | |
| --- | --- | --- | --- |
| Parameters | 0 | 1 | 2 |
| INR | <=1.2 | >1.2 | >1.4 |
| Platelet Count  (x10^9^/L) | >=150 | <150 | <100 |
| SOFA score | 0 | 1 | >=2 |

Total SOFA is the sum the respiratory SOFA, cardiovascular SOFA, hepatic SOFA, and renal SOFA;

Diagnosed as sepsis-induced coagulopathy when the total score >= 4 or sum of the INR score and platelet count score is greater than 2.
