# Supplementary figures and images for: Interpretable machine learning model for early prediction of 28-day mortality in ICU patients with sepsis-induced coagulopathy: development and validation
Source: Eur J Med Res. 2024 Jan 3;29:14. doi: 10.1186/s40001-023-01593-7 (PMC10763177; doi:10.1186/s40001-023-01593-7)

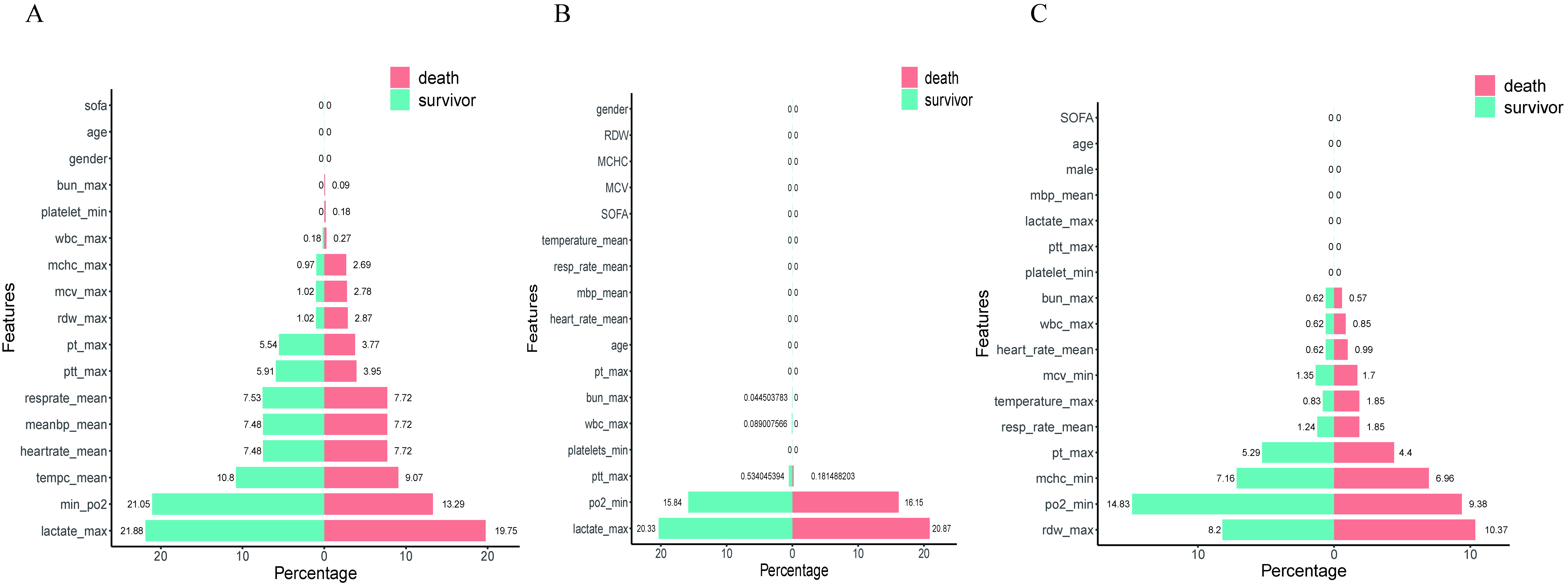

Supplement: Supplementary file 5 — Additional file 5: Figure S2. The detailed comparison of the percentage between missing values for each of the 17 factors between survivors and non-survivors in MIMIC-III (A), MIMIC-IV (B), and eICU-CRD (C) database. [file 40001_2023_1593_MOESM5_ESM.tif]

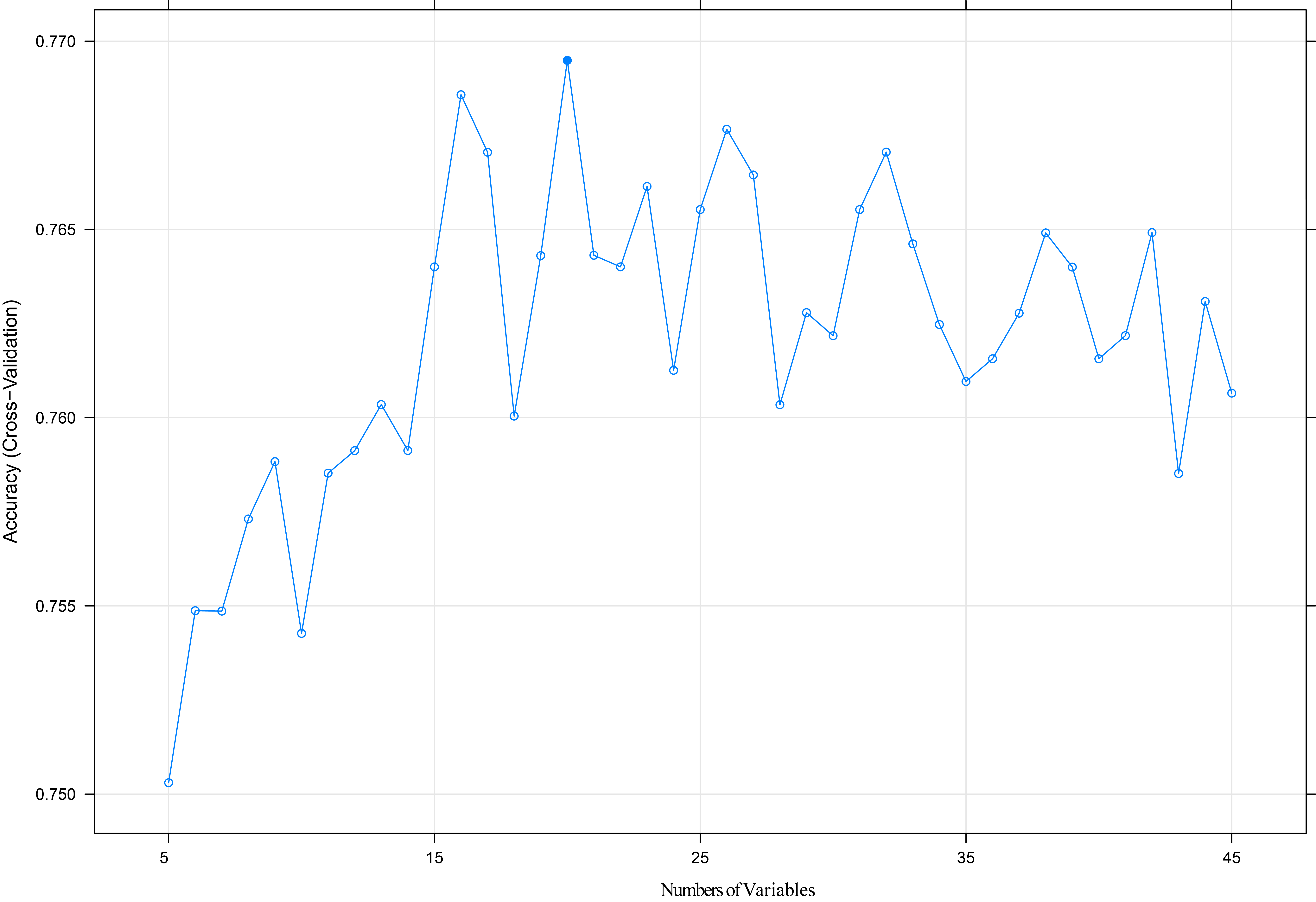

Supplement: Supplementary file 7 — Additional file 7: Figure S4. Feature selection accuracy curve using recursive feature elimination cross-validation. The accuracy get the highest accuracy when the number of variables was 20 (represented as a solid point). [file 40001_2023_1593_MOESM7_ESM.tif]

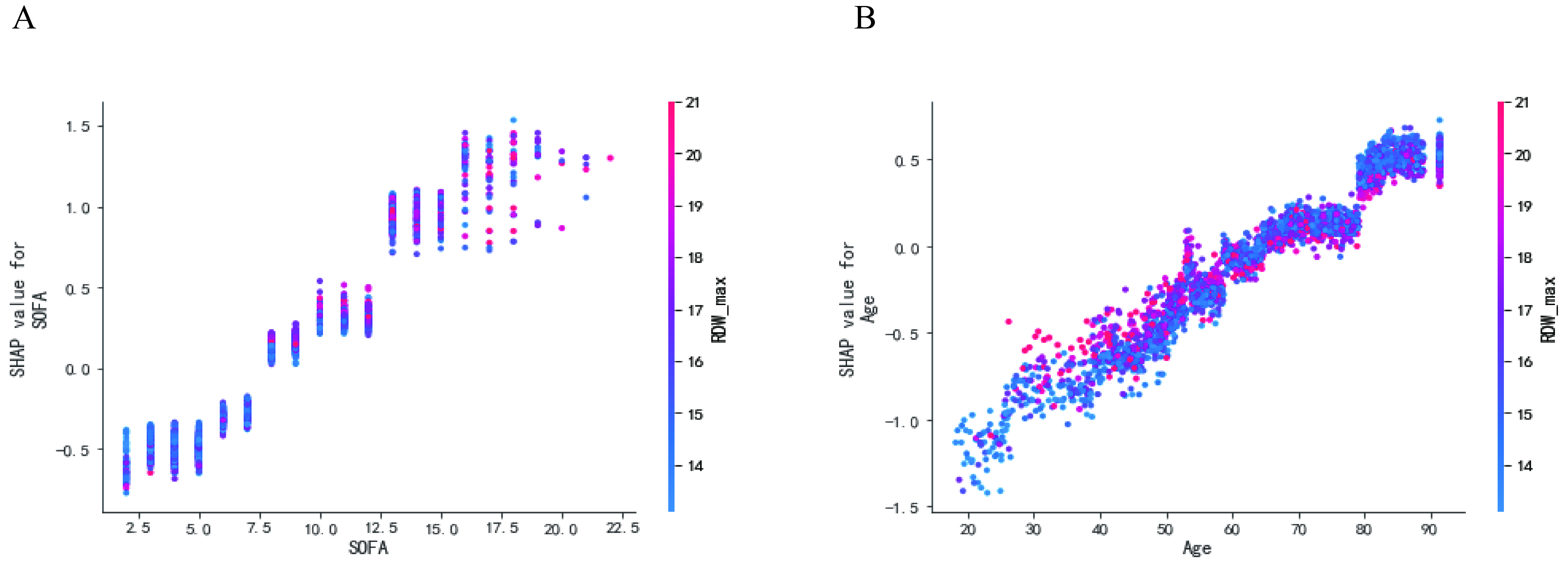

Supplement: Supplementary file 8 — Additional file 8: Figure S5. The potential interactions between RDW with initial SOFA (A) and age (B). The Y-axis on the left represents the SHAP value of SOFA or age, while Y-axis on the right shows the different values of RDW. Despite SOFA or age being identical, the SHAP value corresponding to different RDW levels may be discrepancies. SOFA = sequential organ failure assessment; RDW = red blood cell distribution width. [file 40001_2023_1593_MOESM8_ESM.tif]

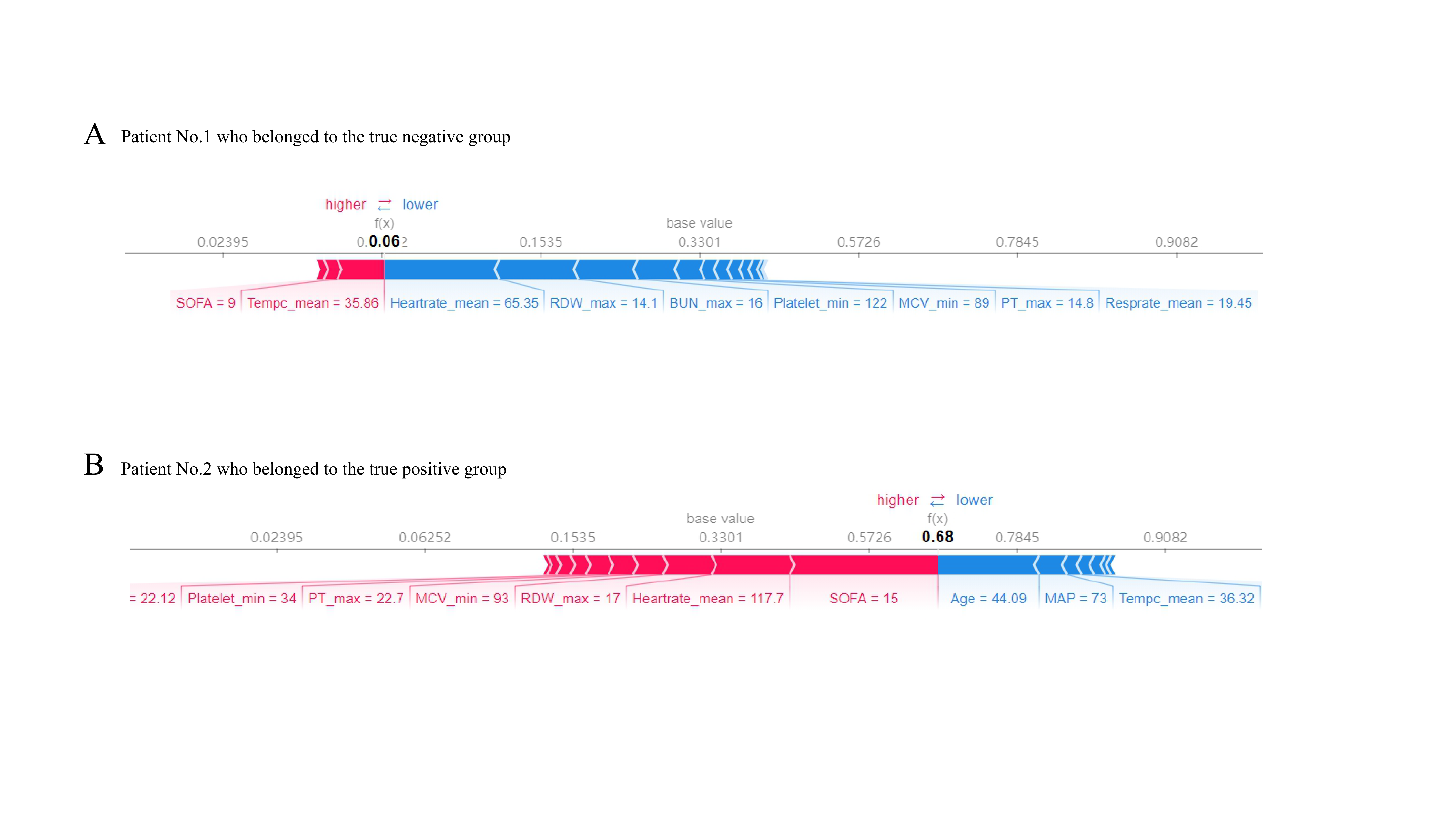

Supplement: Supplementary file 9 — Additional file 9: Figure S6. The interpretation of model prediction results with two actual samples using the SHAP. Patient No.1, who belonged to the "true negative" group, was correctly predicted as a survivor by XGBoost. Patient No.2, who belonged to the "true positive" group, was correctly predicted as a non-survivor. This plot shows significant features contributing to pushing the model output. The blue features decrease the risk of death, while red features promote death. [file 40001_2023_1593_MOESM9_ESM.tif]

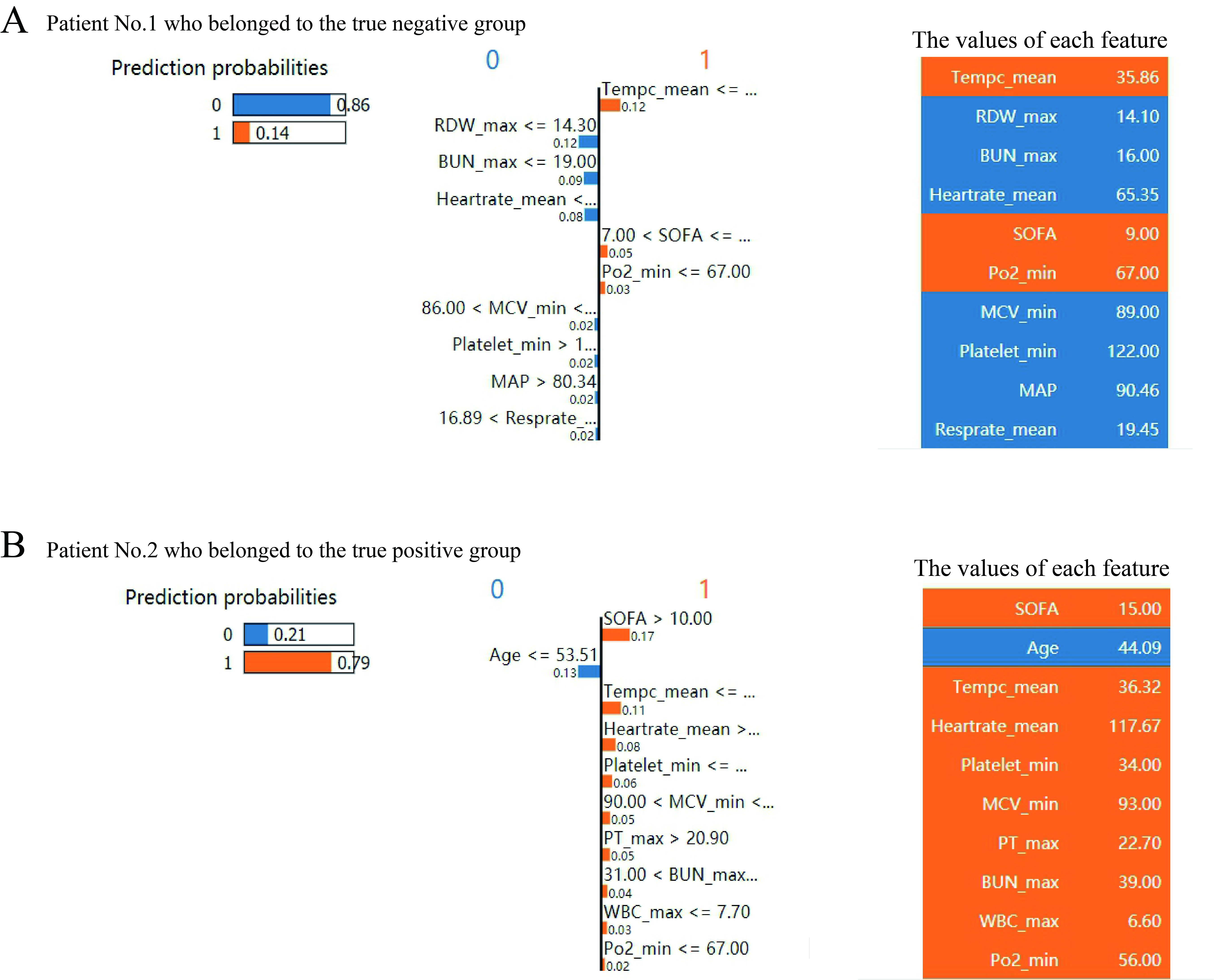

Supplement: Supplementary file 10 — Additional file 10: Figure S7. The interpretation of model prediction results with two actual samples using the LIME. The blue box indicated that the features are risk factors for 28-day death, while the orange box suggests the features are protective factors. [file 40001_2023_1593_MOESM10_ESM.tif]
